# *Zoothamnium mariella* sp. nov., a marine, colonial ciliate with an atypcial growth pattern, and its ectosymbiont *Candidatus* Fusimicrobium zoothamnicola gen. nov., sp. nov.

**DOI:** 10.1371/journal.pone.0300758

**Published:** 2024-04-01

**Authors:** Vincent Kendlbacher, Teresa Maria Rosa Winter, Monika Bright

**Affiliations:** Department of Functional and Evolutionary Ecology, University of Vienna, Vienna, Austria; University of California Irvine, UNITED STATES

## Abstract

Ciliates are unicellular eukaryotes, regularly involved in symbiotic associations. Symbionts may colonize the inside of their cells as well as their surface as ectosymbionts. Here, we report on a new ciliate species, designated as *Zoothamnium mariella* sp. nov. (Peritrichia, Sessilida), discovered in the northern Adriatic Sea (Mediterranean Sea) in 2021. We found this ciliate species to be monospecifically associated with a new genus of ectosymbiotic bacteria, here proposed as *Candidatus* Fusimicrobium zoothamnicola gen. nov., sp. nov. To formally describe the new ciliate species, we investigated its morphology and sequenced its 18S rRNA gene. To demonstrate its association with a single species of bacterial ectosymbiont, we performed 16S rRNA gene sequencing, fluorescence *in situ* hybridization, and scanning electron microscopy. Additionally, we explored the two partners’ cultivation requirements and ecology. *Z. mariella* sp. nov. was characterized by a colony length of up to 1 mm. A consistent number of either seven or eight long branches alternated on the stalk in close distance to each other. The colony developed three different types of zooids: microzooids (“trophic stage”), macrozooids (“telotroch stage”), and terminal zooids (“dividing stage”). Viewed from inside the cell, the microzooids’ oral ciliature ran in 1 ¼ turns in a clockwise direction around the peristomial disc before entering the infundibulum, where it performed another ¾ turn. Phylogenetic analyses assigned *Z. mariella* sp. nov. to clade II of the family Zoothamnidae. The ectosymbiont formed a monophyletic clade within the *Gammaproteobacteria* along with two other ectosymbionts of peritrichous ciliates and a free-living vent bacterium. It colonized the entire surface of its ciliate host, except for the most basal stalk of large colonies, and exhibited a single, spindle-shaped morphotype. Furthermore, the two partners together appear to be generalists of temperate, oxic, marine shallow-water environments and were collectively cultivable in steady flow-through systems.

## Introduction

Symbiosis, the coexistence of organisms belonging to different species in physical contact over an extended period of time [1], is ubiquitous in nature. Many of the intensively studied symbioses are beneficial associations between animal or plant hosts and microbial symbionts, e.g. humans and their gut microbiota [2], legumes and nitrogen-fixing rhizobia [3], the Hawaiian bobtail squid with bioluminescent *Allivibrio fischeri* [4], and the vestimentiferan tubeworms from hydrothermal vents with sulfur-oxidizing, chemoautotrophic bacteria [5–7]. Among unicellular eukaryotes, ciliates are regularly involved in symbiotic associations [8–13], which are fuelled by diverse metabolic reactions [8, 11–17]. Common endosymbionts comprise microalgae and bacteria [8–10]. The well-known marine peniculid ciliate, *Paramecium bursaria*, for example, intracellularly harbors the phototrophic green microalga *Chlorella* [14, 15, 18, 19] as well as bacteria inhabiting various cell compartments [20]. Within the main classes of ciliates, symbionts may colonize not only the inside of host cells, but also the host surface as ectosymbionts [10]. A well-studied ectosymbiosis is that between the peritrichous ciliate *Zoothamnium niveum* and its thiotrophic ectosymbiont *Candidatus* Thiobius zoothamnicola [21–31]. This bacterial symbiont oxidizes sulfide [23, 24] to generate energy for inorganic carbon fixation and subsequently transfer organic carbon to its ciliate host [25]. Besides *Z. niveum*, only few other *Zoothamnium* species are known to be colonized by ectosymbiotic bacteria [32–40].

The genus *Zoothamnium* Bory de St. Vincent, 1824 is assigned to the class Oligohymenophorea de Puytorac et al., 1974, the subclass Peritrichia Stein, 1859, the order Sessilida Kahl, 1933, and the family Zoothamnidae, Sommer 1951 [41]. With more than 70 described species, it is one of the largest and most diverse genera of peritrichous ciliates [42–45]. *Zoothamnium* species are colonial, aquatic organisms, consisting of three different types of zooids, all connected by a common stalk and branches. Dividing terminal zooids, feeding microzooids, and macrozooids capable of leaving the colony as swarmers to settle and build a new colony [31, 46]. On the tip of each colony, a top terminal zooid divides to produce a new terminal zooid (terminal branch zooid), which in turn develops a branch and produces the microzooids of that branch. Thus, the number of branches per colony corresponds to the divisions of the top terminal zooid [23]. A specialised organelle, the spasmoneme, continuously runs inside the stalk and the branches throughout the entire colony, enabling the contraction in a typical zig-zag pattern [41]. Except for the planktonic *Z. pelagicum* [37, 47], all representatives of the genus are sessile, attached to seagrass, macroalgae, and animals as well as various hard substrates and man-made structures. They occur in marine, brackish -, and freshwater environments [41], efficiently filtering their surrounding for microbes and organic particles [48, 49]. New *Zoothamnium* species are continually being described [32, 50–52], suggesting that a great undiscovered diversity exists within the genus.

Here, we report on the discovery of a new *Zoothamnium* species in shallow water environments of the northern Adriatic Sea in 2021. Bacteria covered the surface of the colony, indicating a symbiotic association. We used various microscopic and molecular biological tools, to formally describe this new ciliate species and to demonstrate its monospecific association with a new genus of bacterial ectosymbiont. Additionally, we investigated habitat preferences and cultivation requirements of the two partners.

## Materials and methods

### Ethics statement

Permissions to access the subtidal mudflat Stjuža and the canal Lera were granted by the Strunjan Landscape Park and the Naturepark Sečoveljske soline, respectively. The subtidal sediment in front of the Marine Biology Station Piran is publicly accessible. We confirm that our field study did not include endangered or protected species.

### Sample collection, ecological measurements, cultivation, and fixation

**Fig 1:**
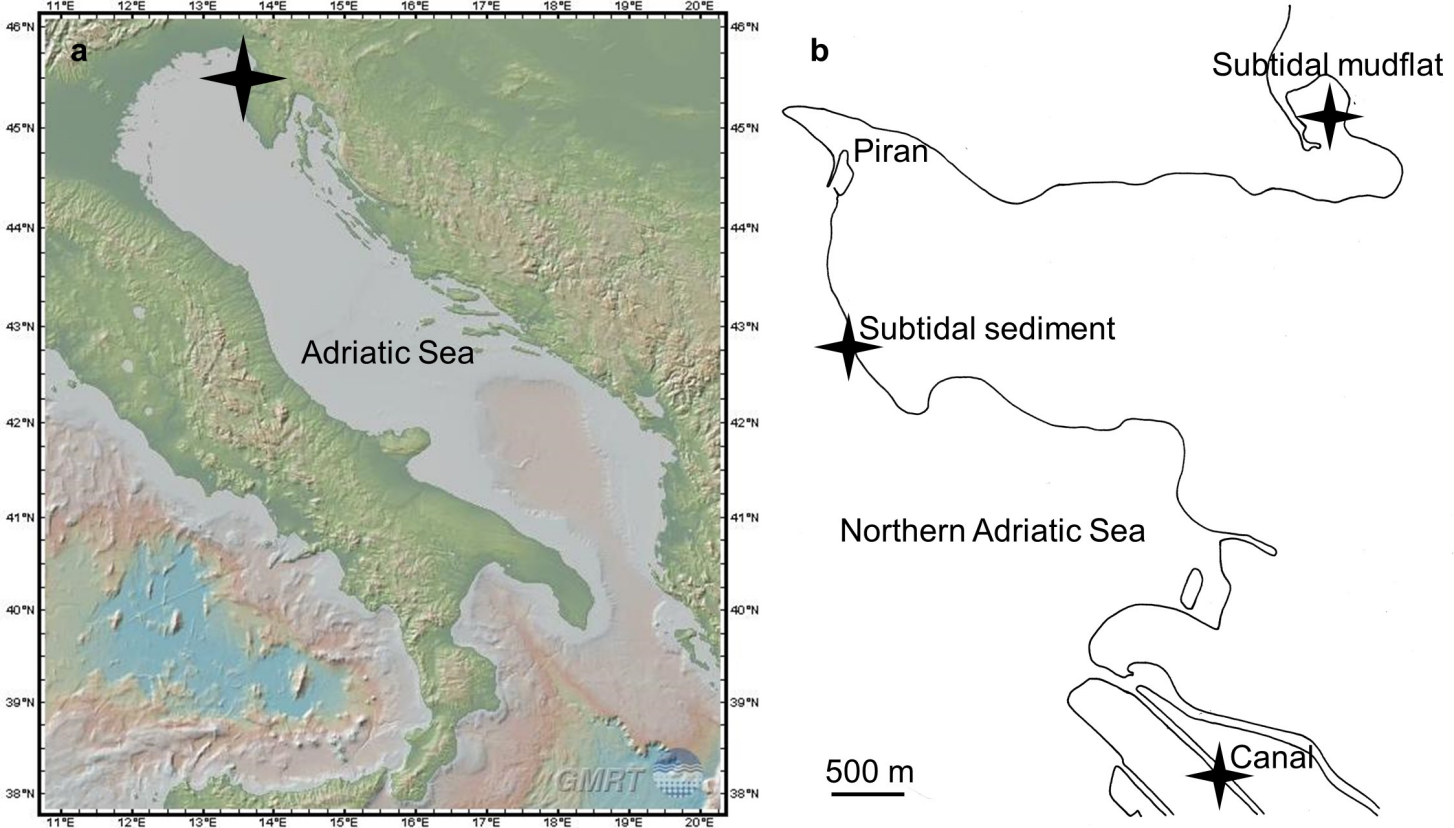
Sampling sites. a) Map showing the Adriatic Sea, reprinted from GeoMapApp (www.geomapapp.org) under a CC BY license, with permission from William B. F. Ryan, original copyright 2009 [53], b) Detailed drawing of the coastline around Piran with special emphasis on the three sampling sites.

We sampled colonies of the new *Zoothamnium* species from wooden- and plastic cubes (12 cm x 11.2 cm x 11.2 cm), that had been deployed in the context of a different project, at three distinct marine sites in the northern Adriatic Sea (Mediterranean Sea) in 2021 and 2022. Beside these experimentally deployed cubes, we sampled further specimens from rocks, macroalgae, dead bryozoans, and cable ties, collected at the same sites. The sites represented the subtidal sediment in front of the Marine Biology Station Piran, the subtidal mudflat Stjuža located in the Strunjan Landscape Park, and the canal Lera extending through the Naturepark Sečoveljske soline (Fig 1). The sampling depth ranged from 1.5 m (subtidal mudflat), over 2.5 m (canal), to 3.5 m (subtidal sediment). Whenever we sampled at one of those sites, we measured the seawater’s temperature and oxygen concentration using a Fibox 4 (PreSens) as well as pH and salinity using a WTW Multi 340i (Wissenschaftlich-Technische Werkstätten).

We cultivated colonies of the new *Zoothamnium* species by placing macroalgae, dead bryozoans, and cable ties, populated by the colonies, underneath an inverted glass container inside a tank with continuous flow of seawater for several days. Between the glass container and the flow-through tank, we left a narrow gap to promote swarmer settlement on the glass surface while allowing water exchange. The seawater that ran through the flow-through tank originated from the subtidal sediment sampling site and exhibited an oxygen concentration of 200 μmol L^-1^, a pH of 8.1, a temperature of 23°C, and salinity values of 36.5.

We fixed and stored colonies for DNA extraction and fluorescence *in situ* hybridization (FISH) alongside colonies designated as type specimens in 100% ethanol. In addition, we fixed colonies for scanning electron microscopy as well as further potential type specimens in a modified Trump’s fixative (2.5% glutaraldehyde, 2% paraformaldehyde in sodium cacodylate 0.1 mol L^-1^; 1100 mOsm L^-1^; pH 7.2) and retained them in the fixative for up to six months until further treatment.

### Microscopic investigations

**Fig 2:**
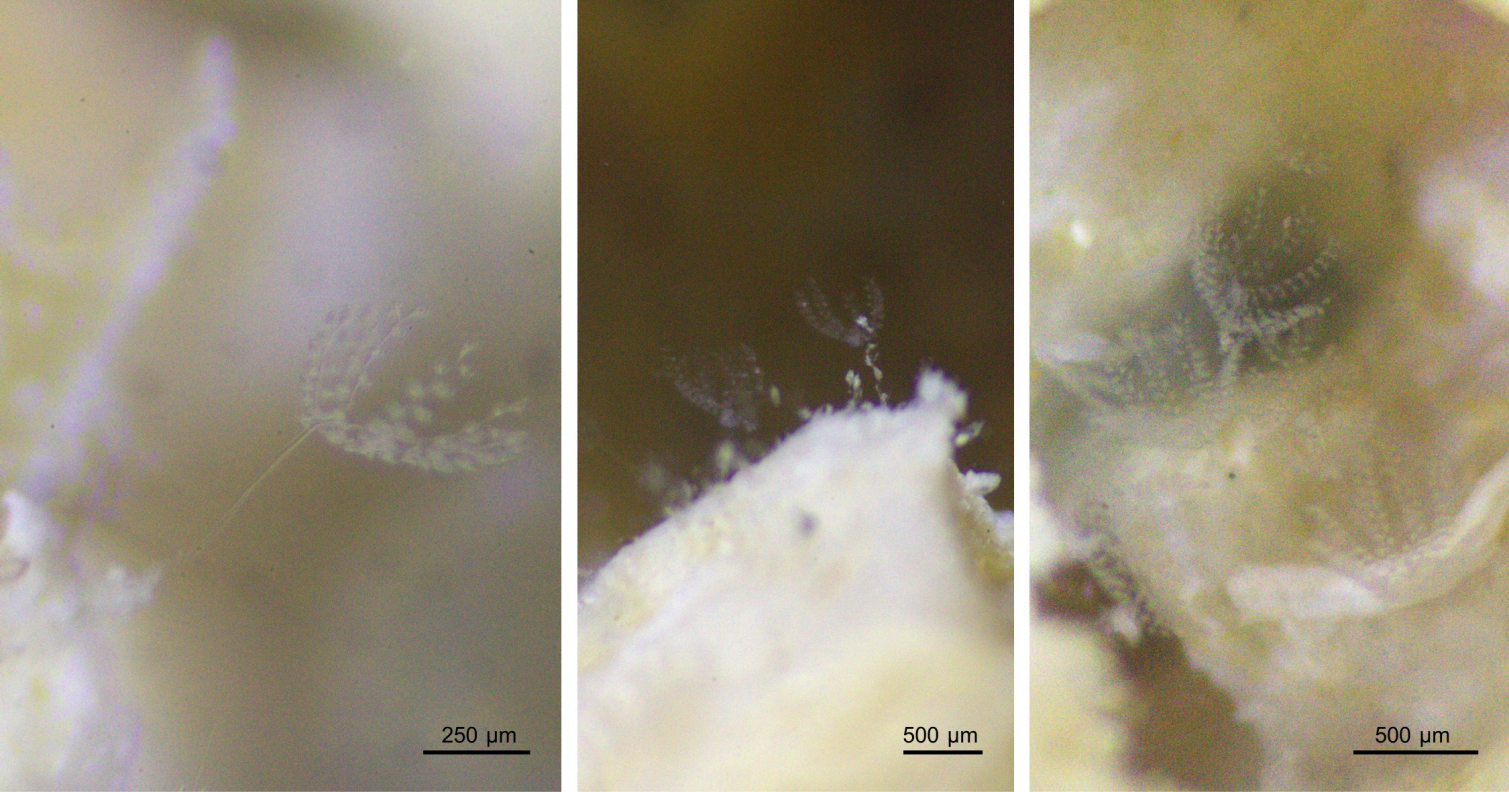
Colonies of *Zoothamnium mariella* sp. nov. attached to wood.

We observed and took micrographs of colonies in vivo, using bright-field and differential interference contrast (DIC) optics on a Leica DM2000 microscope equipped with a Leica DFC295 camera. We used these micrographs to precisely measure entire colonies and individual cells as well as to generate accurate drawings. In order to unveil the kinetosomes, the nuclei and the silverline system, we applied the pyridinated silver carbonate impregnation method, following Fernández-Galiano’s protocol [54], with slight modifications proposed by Wilhelm Foissner [55]. To assess the presence of symbiotic bacteria in colonies of the new *Zoothamnium* species, we employed the LIVE/DEAD BacLight™ Bacterial Viability Kit (Thermo Fisher Scientific) and examined stained colonies using epifluorescence optics (Table 1). Furthermore, we photographed living colonies on wood with a Canon EOS 550D camera on a BMS 144 stereomicroscope (Fig 2).

**Table 1:** Site, substrate, and date of samples used for various methods.

quantity	sampling site	substrate	sampling date	specimen state	application
2	subtidal sediment	wooden cube	March 2021	100% EtOH	18S sequencing
1	subtidal sediment	wooden cube	July 2021	100% EtOH	18S sequencing
2	subtidal mudflat	wooden cube	July 2021	100% EtOH	18S sequencing
7	subtidal sediment	wooden cube	August 2021	100% EtOH	18S sequencing
4	subtidal sediment	wooden cube	February 2022	100% EtOH	18S sequencing
3	subtidal sediment	rock	February 2022	100% EtOH	18S sequencing
1	subtidal mudflat	rock	February 2022	100% EtOH	18S sequencing
2	canal	wooden cube	February 2022	100% EtOH	18S sequencing
1	canal	plastic cube	February 2022	100% EtOH	18S sequencing
4	subtidal sediment	wooden cube	July 2022	100% EtOH	18S sequencing
1	subtidal sediment	plastic cube	August 2022	100% EtOH	18S sequencing
1	subtidal sediment	wooden cube	March 2021	100% EtOH	18S- and 16S sequencing
1	subtidal sediment	wooden cube	July 2021	100% EtOH	18S- and 16S sequencing
1	subtidal mudflat	wooden cube	February 2022	100% EtOH	18S- and 16S sequencing
2	canal	macroalgae	August 2022	100% EtOH	FISH
2	cultivation	glass	August 2022	100% EtOH	FISH
3	canal	dead bryozoans	August 2022	Trump’s fixative	SEM
3	canal	cable ties	August 2022	Trump’s fixative	SEM
3	cultivation	glass	August 2022	Trump’s fixative	SEM
3	canal	wooden cube	August 2022	Trump’s fixative	Type specimens
10	canal	wooden cube	August 2022	100% EtOH	Type specimens
1	cultivation	glass	August 2022	alive	microscopy
1	cultivation	glass	August 2022	alive	Fernández-Galiano
1	canal	wooden cube	August 2022	alive	LIVE/DEAD

### DNA extraction, polymerase chain reaction (PCR) and sequencing

We extracted DNA from 31 colonies using the DNeasy Blood & Tissue Kit (Qiagen) according to the manufacturer’s instructions, except for one minor modification of the reaction volume. Instead of 200 μl elution buffer, we added 100 μl. Subsequently, we amplified the 18S rRNA genes from all 31 DNA extracts by PCR using the universal eukaryotic primers 82 forward [56] and Medlin B reverse [57]. In addition, we amplified the 16S rRNA genes from three DNA extracts employing the general bacterial primers 27 forward and 1492 reverse [58].

To separately clone amplified 16S rRNA genes, we applied the TOPO-TA cloning kit (Invitrogen) according to the manufacturer’s instructions. For each cloning reaction we selected 17 to 18 clones and amplified their inserted 16S rRNA gene by PCR with the M13 forward and M13 reverse primers. Following that, we had both the 18S rRNA genes and the 16S rRNA genes sequenced via Sanger sequencing. We further processed the obtained sequences using Geneious Prime 2022 (https://www.geneious.com) and deposited them in the GenBank database.

### Phylogenetic analyses

We compared the processed 16S and 18S rRNA gene sequences with the National Center for Biotechnology information NCBI (https://www.ncbi.nlm.nih.gov) database utilizing the website’s implemented tool BLAST [59]. To decipher the affiliation of the new *Zoothamnium* species to other species of the genus, we downloaded 18S rRNA gene sequences from NCBI of all members belonging to the monophyletic clade II of the family Zoothamnidae [60]. Concerning phylogenetic analyses of the new ectosymbiont phylotype, we downloaded 16S rRNA gene sequences of BLAST hits with percent identities higher than 90%. Additionally, we added four sequences from the *Z. niveum* symbiont, *Candidatus* Thiobius zoothamnicola, two sequences each from representatives of the order Chromatiales and Methylococcales, and two alphaproteobacterial sequences as an outgroup. We aligned sequences for both genes in SeaView version 5 [61] and checked the alignment manually. Thereafter, we trimmed the sequence alignments to lengths of 1529 base pairs (18S rRNA gene) and 1448 base pairs (16S rRNA gene) using the packages ape version 5.7 [62] and SeqinR version 4.2 [63] in R version 4.1.1 [64]. We trimmed to these specific lengths, because only 10% of the sequences for each gene were shorter. Ultimately, we generated maximum likelihood and maximum parsimony trees with bootstrap support in SeaView version 5.

### Symbiont-specific probe design and fluorescence *in situ* hybridization

We designed two oligonucleotide probes, specific for the new ectosymbiont phylotype, using Geneious Prime 2022. Both probes hybridize to easily accessible regions of the 16S rRNA gene, in order to produce the brightest signals possible [65]. To ensure the probes’ specificity, we compared their sequences with the SILVA database [66] as well as the ribosomal database project applying the website’s tool probematch [67]. Sequences of both probes, termed ZMS152 and ZMS1239, are accessible on probeBase [68]. We labelled ZMS152 at the 3’ end and ZMS1239 at the 5’ end with the fluorescent dye Cy3. Moreover, we embedded four colonies of the new *Zoothamnium* species in LR White resin (London Resin Co.), polymerized at 43°C for three days, and cut semithin sections of 1 μm thickness with a Leica UC7 ultramicrotome. To assess the optimal formamide concentrations for the newly designed probes, we generated a formamide curve utilizing the webtool mathFISH [69]. We ultimately determined the optimal concentrations by increasing formamide in the hybridization reaction from 0 to 30% for ZMS152 and from 0 to 20% for ZMS1239. We hybridized the LR White sections simultaneously with one of the symbiont-specific probes and the EUB mix [70, 71], labelled in Cy5, which served as positive control. Additionally, we added 4’,6-Diamidino-2-phenylindole (DAPI) as a counterstain. For a negative control, we employed a nonsense probe NON338 [72] on individual sections (Table 2). Finally, we investigated the outcome with a Zeiss Axio Imager M2 microscope using epifluorescence optics.

**Table 2:** Probes used for fluorescence *in situ* hybrization.

probe	specificity	sequence	reference
EUB338	most bacteria (EUBI + II + III)	5’-GCT GCC TCC CGT AGG AGT-3’	[70]
EUB338II	most bacteria (EUBI + II + III)	5’-GCA GCC ACC CGT AGG TGT-3’	[71]
EUB338III	most bacteria (EUBI + II + III)	5’-GCT GCC ACC CGT AGG TGT-3’	[71]
NON338	negative control	5’-ACT CCT ACG GGA GGC AGC-3’	[72]
ZMS152	New ectosymbiont phylotype	5’-TNT GCG GTA TTA GCC AAT GTT-3’	this study
ZMS1239	New ectosymbiont phylotype	5’-TCC GGA CTA AGA TCA GAT TTA-3’	this study

### Scanning electron microscopy (SEM)

We rinsed nine colonies, retained in a modified Trump’s fixative, with ultrapure water, to subsequently postfix them in a mixture of 0.1M sodium cacodylate buffer and 1% osmium tetroxide. After rinsing them again with ultrapure water, we dehydrated the colonies in a graded series of ethanol with a final 100% acetone step. Next, we transferred them into 1:1 acetone/hexamethyldisilazane (HMDS) and then into 100% HMDS, where we left them to air-dry. As a final preparation step, we mounted the air-dried colonies on stubs and sputter gold coated them utilizing a JEOL JFC-2300HR. We observed the ready prepared colonies with a JEOL IT 3000 scanning electron microscope.

### Nomenclatural acts

The electronic edition of this article conforms to the requirements of the amended International Code of Zoological Nomenclature, and hence the new names contained herein are available under that Code from the electronic edition of this article. We registered this published work and the nomenclatural acts it contains in ZooBank, the online registration system for the ICZN. The ZooBank LSIDs (Life Science Identifiers) can be resolved and the associated information viewed through any standard web browser by appending the LSID to the prefix “http://zoobank.org/”. The LSID for this publication is: urn:lsid:zoobank.org:pub:57CC07A5-ECED-4FDF-9E3D-FACA53FBBFC3. The electronic edition of this work was published in a journal with an ISSN, and has been archived and is available from the following digital repositories: LOCKSS [author to insert any additional repositories].

## Results

### *Zoothamnium mariella* sp. nov

We followed the classification system proposed by Lynn, 2008 [41].

Phylum: Ciliophora Doflein, 1901

Class: Oligohymenophorea de Puytorac et al., 1974

Subclass: Peritrichia Stein, 1859

Order: Sessilida Kahl, 1933

Family: Zoothamnidae Sommer, 1951

Genus: *Zoothamnium* Bory de St. Vincent, 1824

#### Diagnosis

Star-shaped *Zoothamnium* species up to 1 mm in length with three different types of zooids: microzooids (“trophic stage”), macrozooids (“telotroch stage”), and terminal zooids (“dividing stage”). Colonies developed a final number of either seven or eight long branches that originated alternately on the stalk in close distance to each other. In fully developed colonies, the terminal zooids were restricted to the distal ends of the branches (terminal branch zooids). A terminal zooid on the tip of the stalk (top terminal zooid) only existed in young colonies during colony development. The microzooids were inverted bell-shaped and developed alternately on the branches. The macrozooids exhibited a roundish to ellipsoid shape and were located at the proximal ends of the branches. Undividing terminal zooids resembled the microzooids in shape and size. Dividing terminal zooids became ellipsoid. One or two macrozooids developed per colony. The macronucleus of the microzooids was S-shaped. The macronucleus of the macrozooids appeared thick, constant in diameter, with multiple coils. Viewed from inside the cell, the microzooids’ oral ciliature performed two turns in a clockwise direction. The oral ciliature exhibited a conspicuous curve-shape in the very beginning before initiating its first turn. Species found in marine shallow water environments.

#### Type locality

We collected all type specimens from experimentally deployed wooden cubes at the canal Lera in August 2022 (northern Adriatic Sea; 45° 29’ 46.7” N, 13° 35’ 36.7” E; water depth 2.5 m, water temperature: 26.3°C, salinity: 36.4, pH: 8.1).

#### Type specimens

We embedded the holotype (NHM-EV-5888) and two paratypes (NHM-EV-5889 and NHM-EV-5890), which we had previously fixed in a modified Trump’s fixative, in glycerol, mounted them on microscopic slides, and stored them at the Naturhistorisches Museum (NHM), Wien (Austria). Additionally, we stored ten paratypes (NHM-EV-21536), fixed in 100% ethanol, at the NHM.

#### Gene sequence repository

We deposited the 18S rRNA gene sequence of *Zoothamnium mariella* sp. nov. in the GenBank database under the accession number OR381572.

**Taxonomy. *Zoothamnium mariella*** Kendlbacher, Winter & Bright **sp. nov.** urn:lsid:zoobank.org:pub:57CC07A5-ECED-4FDF-9E3D-FACA53FBBFC3.

#### Etymology

The Latin name ‘Mariella’ translates as star of the sea. We utilized it as a species epithet, treated as a noun in apposition, to emphasize the species’ marine habitat as well as its star-shaped appearance.

#### Description

**Fig 3:**
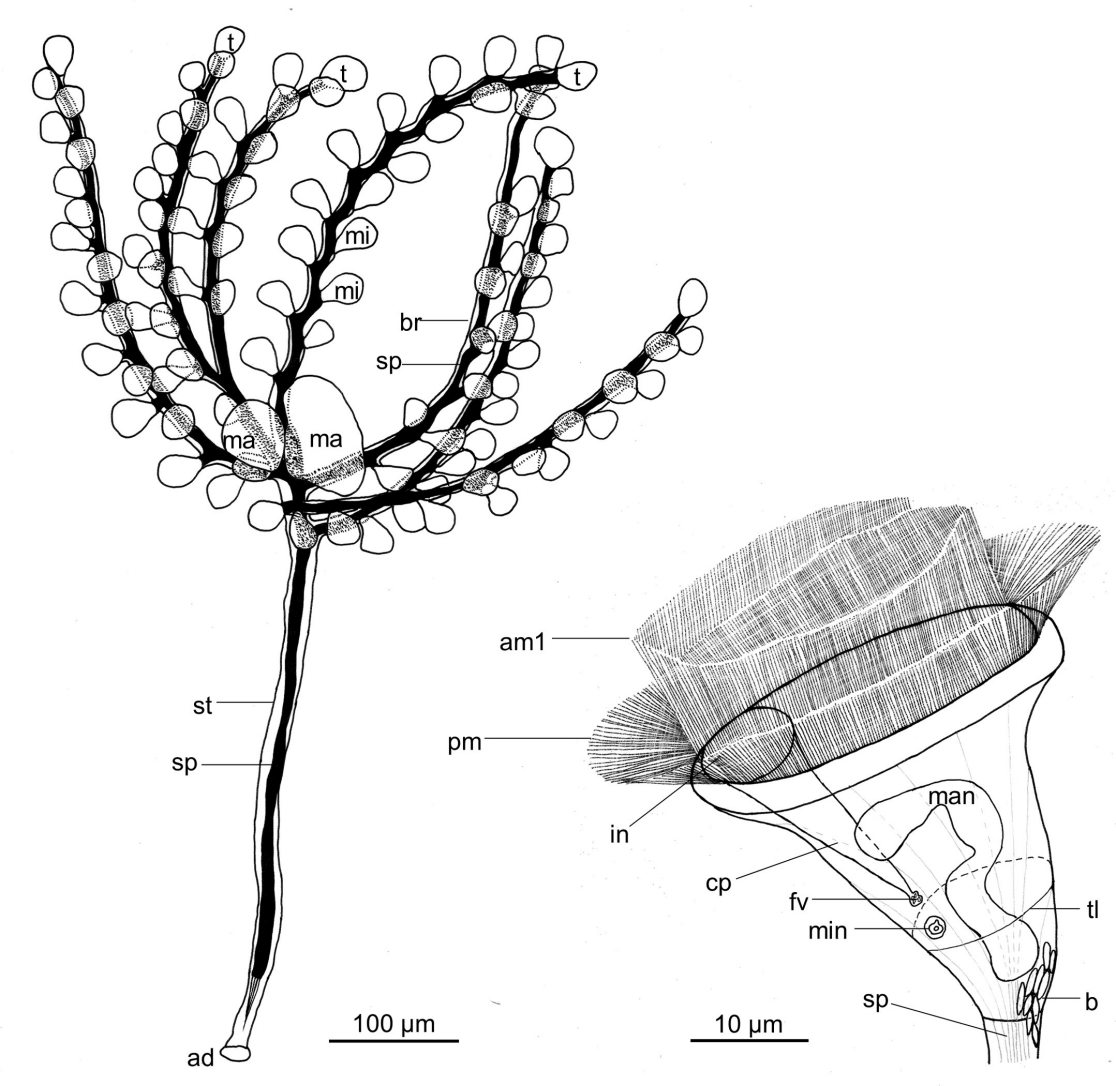
***Zoothamnium mariella* sp. nov**. Detailed drawing of an entire colony and a microzooid, both in expanded condition. mi: microzooid, ma: macrozooid, t: terminal zooid, br: branch, st: stalk, sp: spasmoneme, ad: adhesive disc, am1: adoral membranelle 1 (polykinety), pm: paroral membrane (haplokinety), in: infundibulum, cp: cytopharynx, fv: food vacuole, man: macronucleus, min: micronucleus, tl: telotrochal band, b: bacteria.

**Fig 4:**
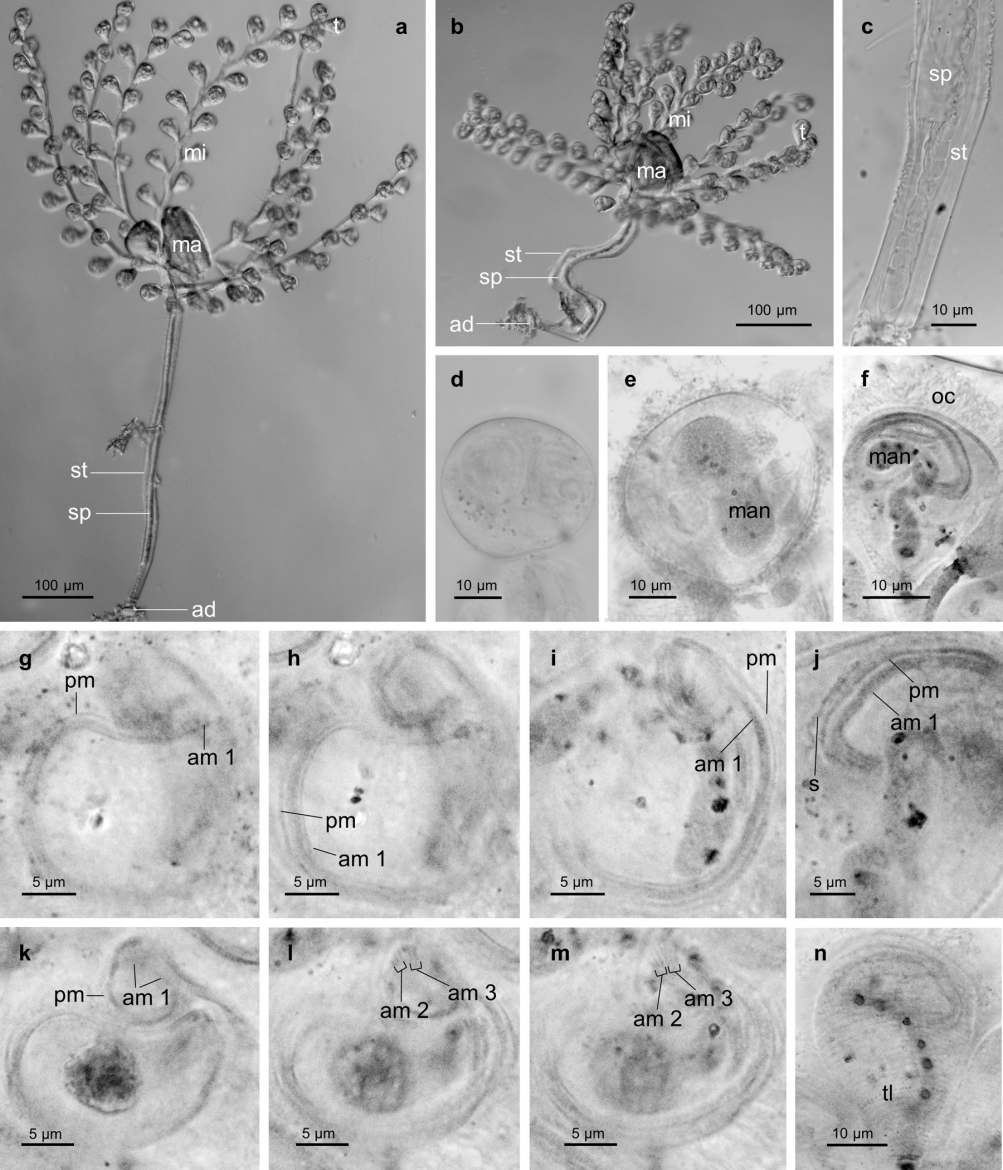
Micrographs of living and silver-stained *Zoothamnium mariella* sp. nov. specimens. a) expanded colony, b) contracted colony, c) most basal part of the stalk, where the spasmoneme splits into bands, which bundle towards the basal end of the colony, d) ellipsoid terminal zooid on the verge of cell division, e) macrozooid, f) microzooid, g–m) oral ciliature of the microzooids, n) microzooid with emphasize on its telotrochal band. a–d) living specimens. e–n) silver-stained specimens. mi: microzooid, ma: macrozooid, t: terminal zooid, st: stalk, sp: spasmoneme, ad: adhesive disc, man: macronucles, oc: oral ciliature, pm: paroral membrane (haplokinety), am1–3: adoral membranelles 1–3 (polykineties), s: stomatogenic kinety, tl: telotrochal band.

The Colony was composed of a stalk with alternately originating branches. Filter-feeding microzooids alternated on the branches. The macrozooids, zooids that leave the colony as swarmers to disperse and build a new colony after settling, occured at the proximal ends of the branches. We observed between one and two macrozooids, with one being up to twice the size of the other. During colony development, the terminal zooid on the tip of the stalk (top terminal zooid) with every division produced a new terminal zooid (terminal branch zooid). Each terminal branch zooid secreted a branch and simultaneously produced the microzooids of that branch. After six to seven divisions, the top terminal zooid ceased to produce further terminal branch zooids, but instead spawned a final branch and the associated microzooids, thereby ending colony growth. Consequently, colonies of *Z. mariella* sp. nov. developed a maximum of either seven or eight branches (Figs 3 and 4).

A specialised organelle, the spasmoneme, ran uninterrupted inside the stalk and branches, extending into every zooid. It facilitated a simultaneous contraction of the entire colony. Additionally, it allowed the oral side of each zooid to contract individually. The basal end of the spasmoneme split into several bands which bundled towards the most basal part of the stalk (Fig 4). Hence, only the most basal part of the stalk and the adhesive disc lacked a spasmoneme. At the basal end of the stalk, the diameter was 15 μm (spasmoneme diameter 10 μm). It increased to 18 μm at the location of the most basal branch (spasmoneme diameter 7 μm) and decreased again to 15 μm (spasmoneme diameter 7 μm) where the top branch originated (S1 Table). The contraction of the colony took place rapidly, while the subsequent expansion was much slower. In the contracted state, the colony showed a typical zigzag pattern (Fig 4). We observed the cilia of the oral apparatus only to beat in the expanded state of the colony. Furthermore, older zooids, attached to further basally located branches, appeared to be less active.

The colony of *Z. mariella* sp. nov. grew up to 1 mm in length. Divided into the following parts ‐ stalk with branches, stalk without branches containing a spasmoneme, and stalk without branches lacking a spasmoneme, the resulting length ratios were 50%, 40% and 10%. The distances between the branches ranged from 15 to 70 μm. The branches exhibited diameters from 9 to 10 μm, with corresponding spasmoneme diameters from 3 to 5 μm. In a full-grown colony, the branches harbored 12 to 18 zooids (distances between zooids 23–30 μm) and reached lengths between 0.29 and 0.43 mm (S1 Table). The length of the branches, their close distance to each other on the stalk, and their small number gave the colony its distinctive star-shaped appearance.

**Fig 5:**
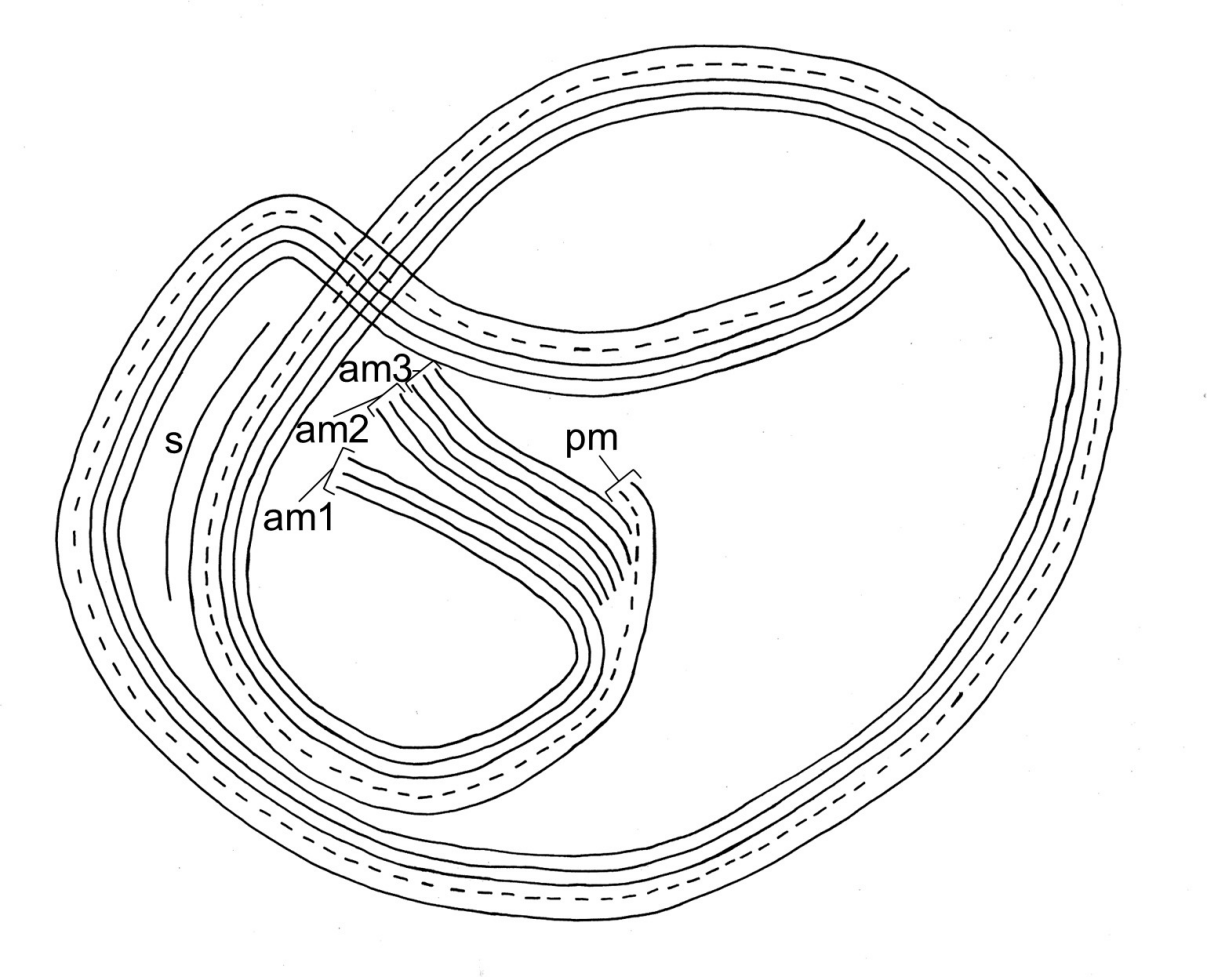
Schematic illustration of the oral ciliature in *Zoothamnium mariella* sp. nov., viewed from the oral pole. pm: paroral membrane (haplokinety), am1–3: adoral membranelles 1–3 (polykineties), s: stomatogenic kinety.

In the expanded colony state with the oral ciliature of the microzooids beating, the microzooids displayed a bulgy, inverted bell-shaped form (average length 23.8 μm, SD 2.6 μm; average oral width 28.3 μm, SD 1.6 μm; average aboral width 6.5 μm, SD 1.0 μm; n = 20; S2 Table; Fig 3). In contrast, contracted microzooids withdrew the whole peristome with the round peristomial disc and the single oral lip, thus appearing more roundish. Orally, the microzooids’ ciliature comprised a paroral membrane (haplokinety), three adoral membranelles (am 1–3; polykineties), and one short stomatogenic kinety (germinal kinety). The paroral membrane was located outside the innermost adoral membranelle (am 1). Viewed from inside the cell, the paroral membrane and the adoral membranelle 1 ran jointly in 1 1/4 turns in a clockwise direction around the peristomial disc. Where they began to run into the infundibulum, a short stomatogenic kinety of barren kinetosomes was present outside the paroral membrane. From there, the paroral membrane and the adoral membranelle 1 extended in another 3/4 turn to the posterior end of the infundibulum, where the adoral membranelle 1 was accompanied by two shorter adoral membranelles (am 2, am3). Furthermore, the paroral membrane and the adoral membranelle 1 exhibited a conspicuous curve-shape in the very beginning before initiating their first turn (Figs 4 and 5).

The microzooids’ pellicula was plain with a striped silverline system (width of the striae 1.0–1.2 μm; S2 Table). Their somatic ciliature was located at about two-thirds distance from the peristomial disc, reduced to a single irregular row of barren kinetosomes, forming the telotrochal band. The macronucleus extended through the entire microzooid and was S-shaped (Figs 3 and 4). Adjacent to the macronucleus, was a single, small, spherical micronucleus.

Expanded terminal zooids showed a bulgy, inverted bell-shaped form, resembling the microzooids in shape and morphological attributes (average length 28.8 μm, SD 3.6 μm; average oral width 28.7 μm, SD 1.5 μm; average aboral width 6.8 μm, SD 1.1 μm; n = 20; S2 Table). Contracted terminal zooids appeared roundish. Furthermore, we occasionally found terminal zooids with an ellipsoid shape (average core width 34.3 μm, SD 5 μm; n = 4; S2 Table) and an enlarged macronucleus, filling up almost the entire cell body (Fig 4). We presumed these zooids to be on the verge of cell division.

The macrozooids were comparably large, roundish to ellipsoid, cells with core widths ranging from 48 to 121 μm (average core width 73.4 μm, SD 23.0 μm; n = 20; S2 Table). Their macronucleus appeared thick, constant in diameter, with multiple coils. Due to its dimensions, it filled up almost the entire cell (Fig 4). The macrozooids’ pellicula had concave bands on its surface, running transverse to the oral-aboral axis of the cell. The width of these striae varied between individual cells and ranged from 0.9 to 3.3 μm (S2 Table). Aborally, a telotrochal band with multiple circular rows of kinetosomes was located at the centre of the cell, arranged like the striae. We observed this telotrochal band to be partly ciliated in attached macrozooids and fully ciliated in free-swimming macrozooids as well as macrozooids about to detach from the colony.

#### 18S rRNA gene sequence and phylogenetic analyses

**Fig 6:**
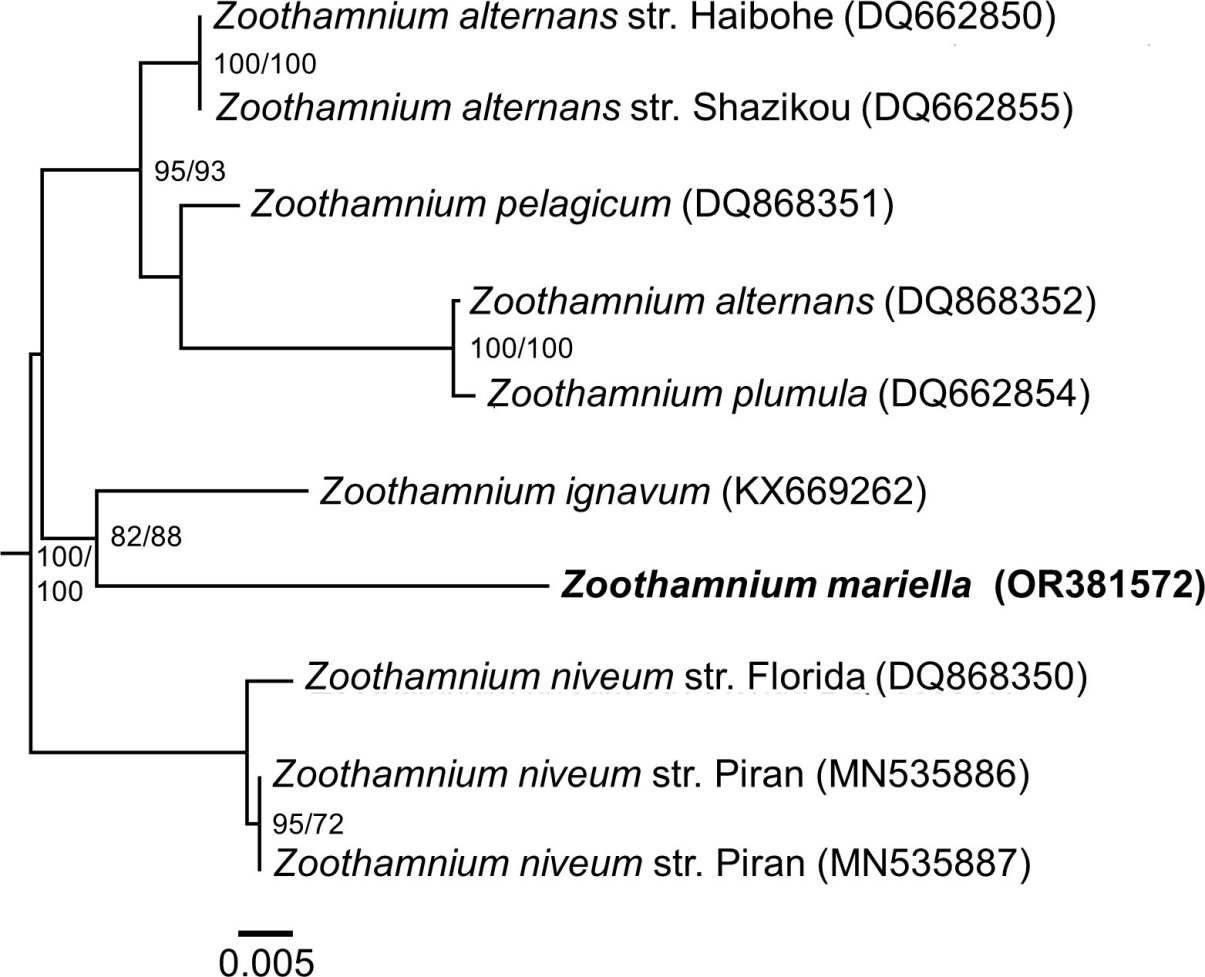
Maximum Likelihood tree inferred from the nucleotide sequences of the small subunit rRNA genes of the monophyletic clade II of Zoothamnidae. The numbers in parentheses are the NCBI accession numbers for each species and the numbers at the nodes represent Maximum Likelihood and Maximum Parsimony bootstrap values greater than or equal to 70%. The scale bar corresponds to 1 substitution per 200 nt positions.

Lengthwise, the 31 processed 18S rRNA gene sequences ranged from 1508 to 1535 base pairs. They exhibited sequence similarities of 100%, indicating that all belonged to the same species. *Zoothamnium mariella* sp. nov. grouped within the monophyletic clade II of the family Zoothamnidae along with *Z. alternans* populations from China and the USA, *Z. ignavum*, *Z. niveum*, *Z. plumula*, and *Z. pelagicum* (Fig 6). With a sequence similarity of 94.67%, the closest relative and sister taxon of *Z. mariella* sp. nov. was *Z. ignavum*. (82% ML, 88% MP).

### *Candidatus* Fusimicrobium zoothamnicola gen. nov., sp. nov

Phylum: *Proteobacteria* Stackebrandt et al., 1988

Class: *Gammaproteobacteria* Garrity et al., 2005

Order: Unclassified

Family: Unclassified

#### Type locality

Same as for the host species *Zoothamnium mariella* sp. nov.

#### Gene sequence repository

We deposited the 16S rRNA gene sequence of *Candidatus* Fusimicrobium zoothamnicola gen. nov., sp. nov. in the GenBank database under the accession number OR387482.

#### Etymology

The generic name Fusimicrobium is a combination of the latin word ‘fusus’ (spindle) and ‘microbium’ (microbe) and highlights the microbe’s spindle-shaped appearance. The species epithet zoothamnicola translates as inhabitant of *Zoothamnium* and refers to the species’ symbiotic association with *Zoothamnium mariella* sp. nov.

#### 16S rRNA gene sequence and phylogenetic analyses

**Fig 7:**
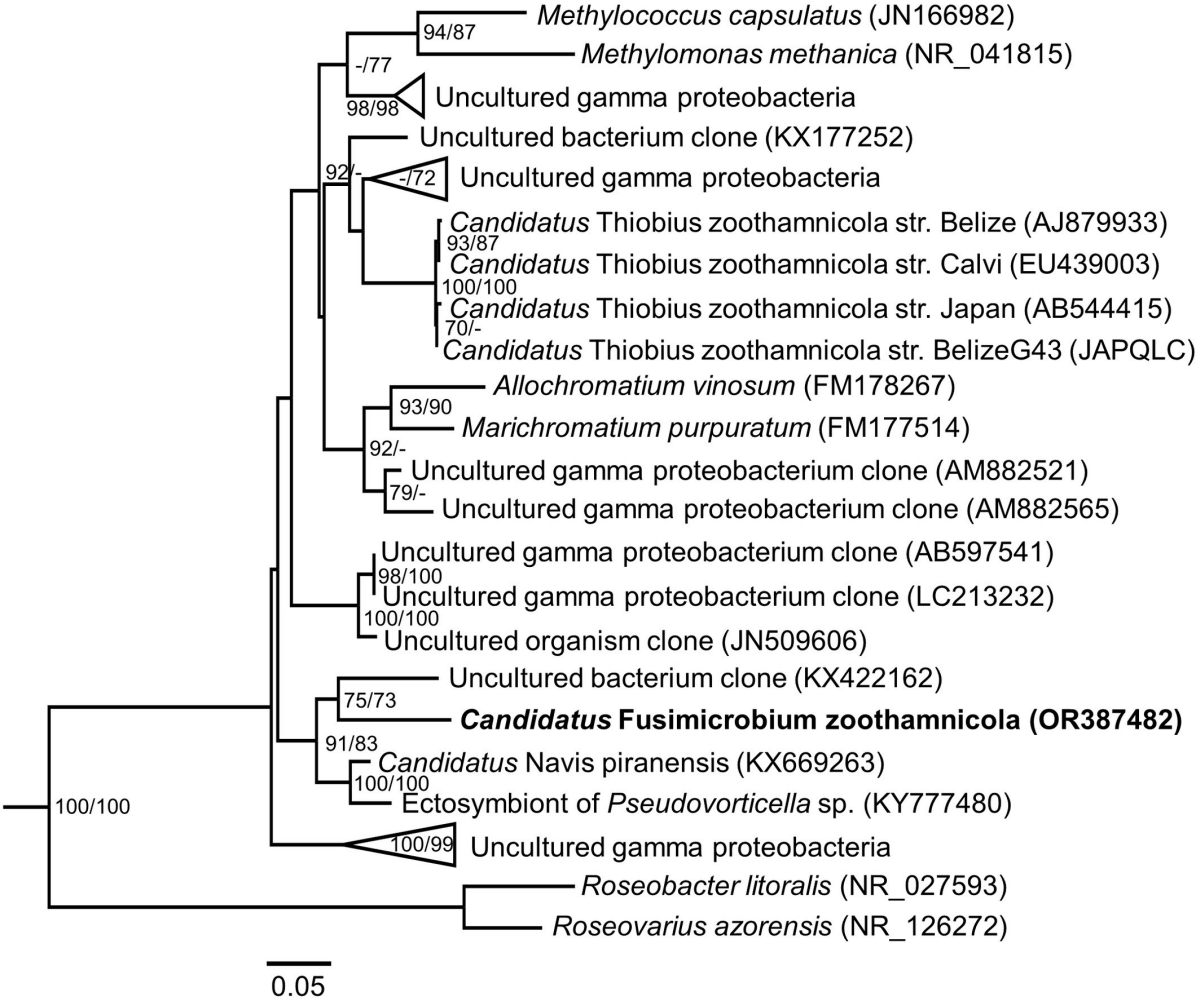
Maximum Likelihood tree inferred from the nucleotide sequences of gamma proteobacterial small subunit rRNA genes displaying the phylogeny of *Ca*. Fusimicrobium zoothamnicola gen. nov., sp. nov. The numbers in parentheses are the NCBI accession numbers for each species and the numbers at the nodes represent Maximum Likelihood and Maximum Parsimony bootstrap values greater than or equal to 70%. The scale bar corresponds to 5 substitutions per 100 nt positions.

From a total of 52 screened clones, 36 carried the 16S rRNA gene sequence belonging to *Candidatus* Fusimicrobium zoothamnicola gen. nov., sp. nov. These 36 sequences exhibited a length of 1457 base pairs and sequence similarities between 99.36 and 100%. The remaining 16 clones contained 16S rRNA gene sequences from different random marine bacteria. Phylogenetic analyses revealed that *Ca*. Fusimicrobium zoothamnicola gen. nov., sp. nov. belongs to the class of *Gammaproteobacteria*. Within this class, it formed a monophyletic clade with the thiotrophic ectosymbiont of *Pseudovorticella* sp. (Peritrichia, Sessilida, Vorticellidae), the ectosymbiont of *Zoothamnium ignavum Candidatus* Navis piranensis, and a free-living bacterium associated with intertidal hydrothermal vents from southern California (Fig 7). With a 16S rRNA gene similarity of 91.36%, the closest relative and sister taxon of *Ca*. Fusimicrobium zoothamnicola gen. nov., sp. nov. turned out to be the free-living vent bacterium. Moreover, *Z. niveum*’s ectosymbiont *Ca*. Thiobius zoothamnicola proved to be only distantly related.

#### Fluorescence *in situ* hybridization (FISH)

**Fig 8:**
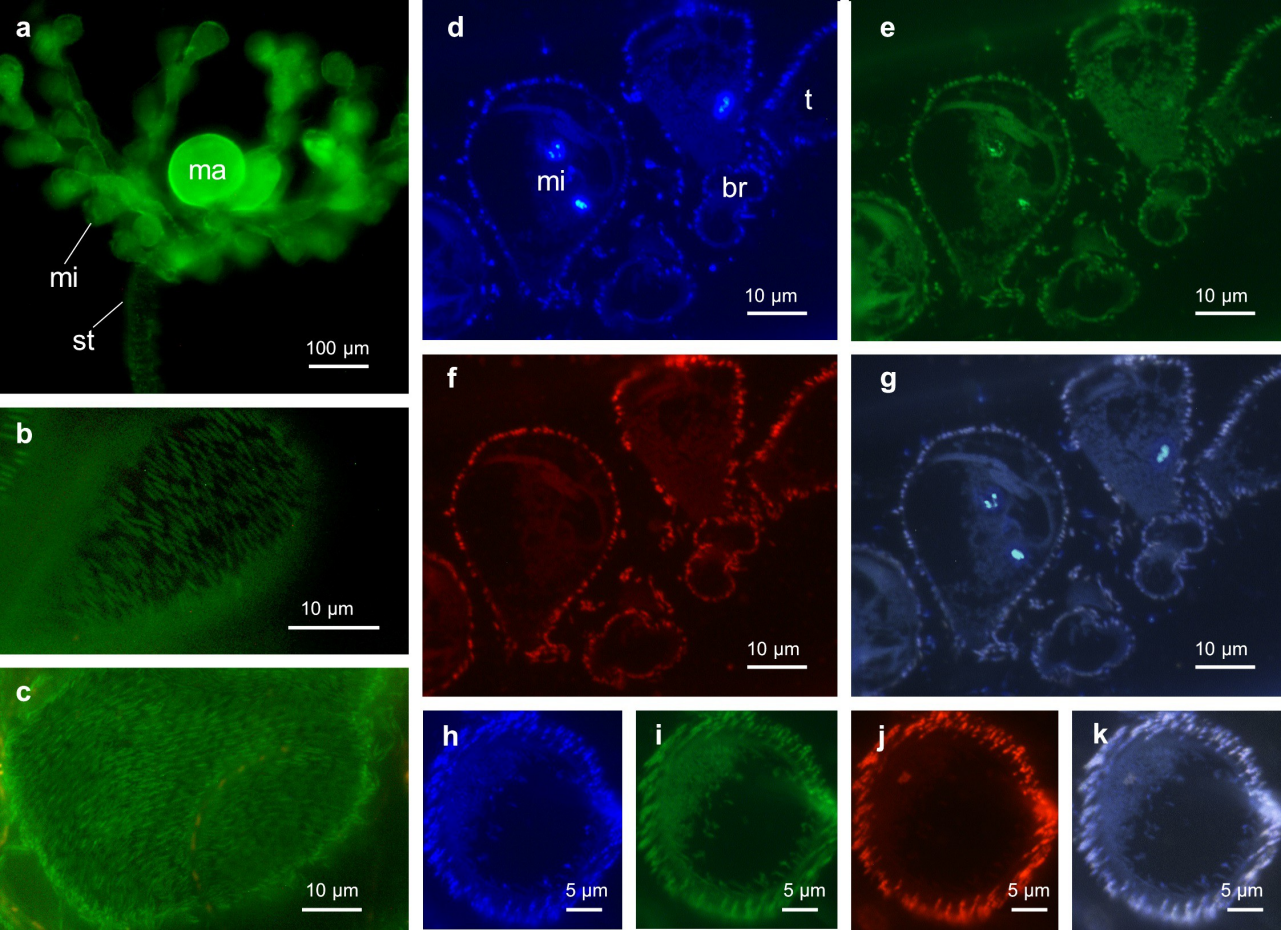
Epifluorescent micrographs showing the *Z. mariella* sp. nov. symbiosis. a, b, c) LIVE/DEAD staining, d,h) DAPI (blue), e, i) EUB mix in Cy5 (green), f, j) probe ZMS152 and ZMS1239 in Cy3 (red), g, k) overlay of the three colours, mi: microzooid, ma: macrozooid, t: terminal zooid, st: stalk, br: branch.

The two newly designed FISH probes, ZMS152 and ZMS1239, both showed at least one mismatch to all other 16S rRNA gene sequences available in the public databases. Performing FISH with the two probes resulted in bright signals which confirmed that *Ca*. Fusimicrobium zoothamnicola gen. nov., sp. nov., indeed, is the ectosymbiont of *Zoothamnium mariella* sp. nov. Optimal formamide concentrations in the hybridization buffer proved to be 20% for ZMS152 and 10% for ZMS1239, precisely matching the formamide curve generated by mathFISH. Generally, all bacteria inhabiting the surface of *Z. mariella* sp. nov. hybridized with the symbiont-specific probes as well as the EUB mix probe set, demonstrating that no additional bacteria contributed to the bacterial coat. The ectosymbionts colonized the surfaces of microzooids, terminal zooids, macrozooids, branches, and stalk (Fig 8). In large colonies, we occasionally found the basal part of the stalk to be overgrown by various unspecific bacteria (S1 Fig) which hybridized solely with the EUB mix probe set. Performing FISH with the nonsense probe NON338 did not yield a detectable fluorescence signal, indicating that all signals originated from specific binding of the probes rather than unspecific staining or autofluorescence.

#### Scanning electron microscopy (SEM)

**Fig 9:**
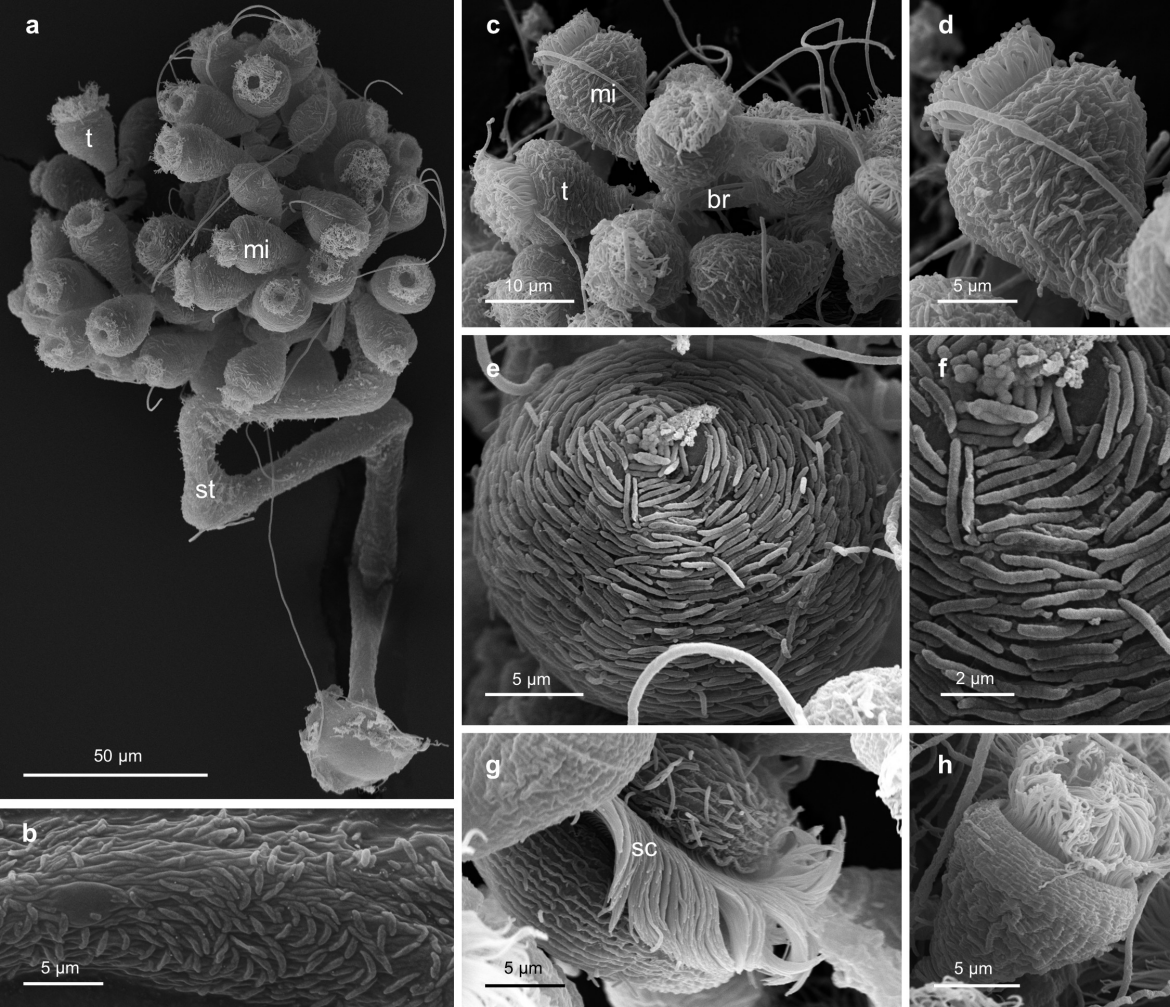
Scanning electron micrographs of *Zoothamnium mariella* sp. nov. a) entire colony in contracted state, b) stalk with bacterial coat, c) branch with microzooids and a terminal zooid, d) microzooid, e) macrozooid, f) bacteria colonizing macrozooid, g) barely colonized macrozooid, h) barely colonized microzooid. mi: microzooid, t: terminal zooid, st: stalk, br: branch, sc: somatic ciliature.

During the fixation process for SEM, apparently the colonies have partly lost their ectosymbionts. Living specimens, either viewed with DIC optics (S2 Fig) or treated with the LIVE/DEAD staining kit (Fig 8), or ethanol-fixed specimens used for FISH (Fig 8), showed a consistent bacterial coat throughout. In contrast, colonies prepared for and observed by SEM were only fragmentarily covered by bacteria (Fig 9). This fragmented bacterial coat consisted exclusively of spindle-shaped cells (average length 2.4 μm, SD 0.6 μm; average width 0.4 μm, SD 0.1 μm; n = 20; S3 Table) and extended from the basal part to the top end of the stalk, over the branches, and onto all three types of zooids. On the stalk, the branches, the microzooids, and the terminal zooids, we found the symbionts arranged in a monolayer. On the macrozooids they appeared to overlap in a multilayered fashion, with not every symbiont cell in direct contact with the host (Fig 9). We could not determine, however, whether this was a further artifact caused by the fixation. At an average length of 2.6 μm (SD 0.2 μm; n = 14; S3 Table) the symbionts initiated binary fission, forming two equally long daughter cells.

### Ecology of the symbiosis

We encountered the symbiosis on various substrates (sunken wood, plastics, dead bryozoans, macroalgae, shells, and rocks) at all three sampling sites during summer and winter seasons. Consequently, both partners were exposed to changing biotic factors as well as physicochemical conditions of the surrounding seawater. We measured oxygen concentrations ranging from 168 to 267 μmol L^-1^, a pH between 8.1 and 8.3, a temperature range of 8°C to 27°C, and salinity values varying between 34.1 and 36.8. Whenever we found the symbiosis on sunken wood, it colonized parts of the wood that showed no signs of leaking sulfide, a chemical produced by anaerobic, sulfate-reducing bacteria during wood degradation [73].

The cultivation experiment under oxic, flow-through conditions, resulted in dense populations of symbiotic *Zoothamnium mariella* sp. nov. populating the surface of the glass container. In addition, we found two other *Zoothamnium* species colonizing the glass surface, *Z. ignavum* and a so far undescribed species. However, both species appeared in low numbers, compared to *Z. mariella* sp. nov.

## Discussion

### The host *Zoothamnium mariella* sp. nov

We identified *Zoothamnium mariella* sp. nov. as a member of the genus *Zoothamnium* due to its sessile, colonial appearance, with individual zooids connected by a common stalk and branches. Furthermore, stalk and branches were internally traversed by a continuous spasmoneme which extended into every zooid and enabled the entire colony to contract in a typical zig-zag pattern. The conspicuous morphology of the colony resulted from the peculiar behaviour displayed by the terminal zooid on the tip of the stalk (top terminal zooid) during colony development. As in any other species of the genus [31, 32, 37, 74–76], it produced with every division a new terminal zooid (terminal branch zooid), which in turn secreted a branch and simultaneously produced the microzooids of that branch. However, in *Z. mariella* sp. nov. the top terminal zooid ceased to produce further terminal branch zooids after six to seven divisions. Instead, it spawned a final branch and the associated microzooids, thus ending colony growth. Consequently, colonies of *Z. mariella* sp. nov. developed a final number of either seven or eight branches. As the branches originated from the stalk in close distance (15–70 μm) to each other, we suspect that the divisions of the top terminal zooid occur at short intervals. Additionally, with a length of up to 0.43 mm, the branches appeared very long, compared to the colony’s total length of about 1 mm.

A similar growth pattern does not exist in any other clade II *Zoothamnium* species [60]. In colonies of *Z. niveum* Ehrenberg, 1838, *Z. ignavum* Schuster & Bright, 2016, *Z. alternans* Claparède & Lachmann, 1859, *Z. plumula* Kahl, 1933 as well as individual colonies of *Z. pelagicum* Du Plessis, 1891 pseudocolonies, the top terminal zooid remained at the tip of the stalk until colony death. It did not transform into a terminal branch zooid after a certain number of cell divisions, which would prevent the colony from developing further branches. Therefore, none of the species has been reported to grow a consistent, maximum number of branches [21, 31–33, 37, 47, 75–81].

Besides this difference, the colony of *Z. mariella* sp. nov. showed morphological similarities to *Z. ignavum* and *Z. alternans* that could potentially lead to confusion between the species. When dividing a *Z. mariella* sp. nov. colony in two parts, stalk with branches, and stalk without branches, the resulting relative lengths were 50% and 50%. This ratio resembled a lot the one displayed by *Z. ignavum* colonies, which was 40% and 60%. However, in *Z. mariella* sp. nov. 80% of the stalk without branches was traversed by a spasmoneme, compared to only 50% in *Z. ignavum*. With a maximum colony length of 1 mm, *Z. mariella* sp. nov. was slightly shorter than the 1.8 mm long *Z. ignavum*. Nevertheless, it grew longer branches. The longest branches in *Z. ignavum* colonies harbored about 14 zooids. In *Z. mariella* sp. nov. we counted up to 18 zooids attached to a single branch, with distances between the zooids corresponding to those of *Z. ignavum* [32]. The possibility of confusing *Z. mariella* sp. nov. and *Z. alternans* derives from their similar size. At 1.2 mm, *Z. alternans* was merely 0.2 mm longer than *Z. mariella* sp. nov. However, in *Z. alternans* the part of the stalk with branches made up 80% of the entire colony, as opposed to 50% in *Z. mariella* sp. nov. With a mean length of 48.4 μm, *Z. alternans*’ microzooids were around twice as long as the average 23.8 μm long microzooids of *Z. mariella* sp. nov. Also, they had a J-shaped macronucleus, whereas in microzooids of *Z. mariella* sp. nov. it was S-shaped [33, 78].

*Z. mariella* sp. nov, *Z. ignavum*, and *Z. alternans* can further be differentiated by investigating their oral ciliature. The paroral membrane and the adoral membranelle 1 of *Z. mariella* sp. nov. and *Z. ignavum* began with 1 1/4 turns around the peristomial disc. From there, they extended into the infundibulum performing another 3/4 turn in *Z. mariella* sp. nov. and 1/4 turn in *Z. ignavum*. In *Z. alternans*, the paroral membrane and the adoral membranelle 1 made 1 1/3 turns around the peristomial disc before entering the infundibulum, where they performed another full turn (Table 3). Furthermore, the paroral membrane and the adoral membranelle 1 of *Z. mariella* sp. nov. exhibited a conspicuous curve-shape in the very beginning, neither present in *Z. ignavum* nor in *Z. alternans* [32, 33].

**Table 3:** Comparison between *Zoothamnium mariella* sp. nov, *Z. ignavum*, and *Z. alternans*.

		*Z. mariella* sp. nov.	*Z. ignavum*	*Z. alternans*
**colony** (S1 Table)	max. size [mm]	1	1.8	1.2
	st + br + sp [%]	50	40	80
	st ‐ br + sp [%]	40	30	10
	st ‐ br–sp [%]	10	30	10
	branches	7–8	at least 22; variable	at least 19; variable
**microzooids** (S2 Table)	length [μm]	23.8 ± 2.6	39.4 ± 3	48.4 ± 4.9
	oral width [μm]	28.3 ± 1.6	28.8 ± 3.1	29.3 ± 1.7
	aboral width [μm]	6.5 ± 1	8.2 ± 1.9	-
	oral ciliature	1 ¼ turns around the peristomial disc, infundibular kineties perform another ¾ turn	1 ¼ turns around the peristomial disc, infundibular kineties perform another ¼ turn	1 1/3 turns around the peristomial disc, infundibular kineties perform another full turn
	macronucleus	S-shaped	S-shaped	J-shaped
**habitat**		marine	marine	marine
**reference**		this study	[21, 32]	[33]

st + br + sp: stalk with branches and spasmoneme; st ‐ br + sp: stalk without branches but with spasmoneme; st ‐ br ‐ sp: stalk without branches and spasmoneme

Molecular phylogenetic analyses based on the 18S rRNA gene sequence revealed that *Z. mariella* sp. nov. belongs to clade II of the family Zoothamnidae [60], with *Z. ignavum* being the sister taxon (94.67% sequence similarity). Within the six members of this clade, at least five form an ectosymbiotic association with bacteria, from which three are known to be monospecific. These are the symbioses between *Z. niveum* and the thiotrophic bacterium *Ca*. Thiobius zoothamnicola [23, 25–28, 31], *Z. ignavum* and *Ca*. Navis piranensis [32] as well as *Z. mariella* sp. nov. and *Ca*. Fusimicrobium zoothamnicola gen. nov., sp. nov. [this study]. Furthermore, *Z. alternans’* pellicula has been observed to be populated by yet unknown bacteria [33–35] and *Z. pelagicum* has ectosymbionts presumed to be cyanobacteria [36–39]. Only in *Z. plumula* symbionts were not mentioned [81, 82]. The tendency of clade II *Zoothamnium* species to form ectosymbioses with bacteria, suggests that their common ancestor has evolved the ability to establish such associations.

### The symbiont *Candidatus* Fusimicrobium zoothamnicola

Microscopic investigations and molecular biological analyses demonstrated that a single species of bacteria, *Candidatus* Fusimicrobium zoothamnicola gen. nov., sp. nov., forms an ectosymbiotic association with *Zoothamnium mariella* sp. nov. These bacteria densely populated the stalk, the branches, the macrozooids, the microzooids, and the terminal zooids.

The 16S rRNA gene sequence assigned the ectosymbiont *Ca*. Fusimicrobium zoothamnicola gen. nov., sp. nov to a monophyletic clade within the *Gammaprotoebacteria*. Also, the ectosymbiont of *Z. ignavum Ca*. Navis piranensis, the thiotrophic ectosymbiont of *Pseudovorticella* sp. (Peritrichia, Sessilida, Vorticellidae), and a free-living bacterium associated with intertidal hydrothermal vents from southern California belonged to this clade. The ectosymbionts of the two host sister taxa, *Z. mariella* sp. nov and *Z. ignavum*, both from the northern Adriatic Sea, however, were not found to be sister taxa themselves. Instead, the sister taxon of *Ca*. Fusimicrobium zoothamnicola gen. nov., sp. nov. turned out to be the free-living vent bacterium [83]. With a sequence similarity of merely 91.36%, *Ca*. Fusimicrobium zoothamnicola gen. nov., sp. nov. can not only be assigned to a new species [84], but also to a new genus [85]. The sister taxon of *Ca*. Navis piranensis, was the thiotrophic ectosymbiont of *Pseudovorticella* sp. collected from degrading mangrove leaves in Guadeloupe [86]. As far as is known, transmission in *Zoothamnium* species is vertical from one host generation to the next, since macrozooids that leave the mother colony to settle and build a new colony are covered with the symbionts [22, 31, 32, 46]. Nevertheless, there was no congruence between host and symbiont phylogeny. This strongly indicates that hosts and symbionts did not co-diversify but that ectosymbiosis has evolved in this genus de novo several times.

At the basal part of the stalk of large *Z. mariella* sp. nov. colonies, we, occasionally, found the monospecific bacterial coat to be replaced by various, unspecific bacteria. Similar nonspecific epigrowth occurred in *Z. ignavum* [32] and *Z. niveum* [26, 31]. Rinke et al., 2006 suspected the initiation of senescence at the most basally located parts of old colonies to be responsible for the loss of the specific ectosymbionts. They hypothesized that as the host structures become senescent, the specific ectosymbionts may detach and are subsequently replaced by other microbes, indicating a host-induced mechanism to retain the monospecific bacterial coat [26].

In the *Z. mariella* sp. nov. symbiosis, the bacterial coat comprised only a single, spindle-shaped morphotype of *Ca*. Fusimicrobium zoothamnicola gen. nov., sp. nov. This morphological uniformity suggests that the nutritional supply for the ectosymbionts is similar regardless of location on the host, because cell form modulations in bacteria are considered to be exclusively related to nutrition [87]. Symbiotic bacteria that develop into distinct morphotypes are a widespread phenomenon, e.g. the endosymbionts of the vestimentiferan species like *Riftia pachyptila* [88, 89]. Similarly, the ectosymbionts of *Z. ignavum* and *Z. niveum* transitioned between two morphotypes. At the oral region of the microzooids, they were coccoid-shaped, on all other parts of the host, they appeared as rods [26, 32]. In the thiotrophic *Z. niveum* symbiosis, for example, it was shown that the coccoid-shaped symbionts at the oral region of the microzooids indeed are exposed to a favorable physicochemical microenvironment [29, 30]. In cultivation experiments where all symbionts had equal access to electron donor and acceptor, the morphological differences between them vanished [23].

### Ecology of the symbiosis

We found this new symbiosis between *Zoothamnium mariella* sp. nov. and *Candidatus* Fusimicrobium zoothamnicola gen. nov., sp. nov. in three different shallow water habitats in the northern Adriatic Sea, a subtidal sediment, a subtidal mudflat, and a canal, on various substrates (sunken wood, plastics, dead bryozoans, macroalgae, shells, and rocks), in summer and in winter. It tolerates at least temperatures ranging from 8°C to 27°C as well as oxygen concentrations between 168 and 267 μmol L^-1^ (oxygen saturation 82% at 27°C and 91% at 8°C). On sunken wood, we encountered the symbiosis solely on parts of the wood that showed no signs of leaking sulfide. All these observations collectively suggest that: (1) the two partners together are generalists of oxic, temperate, shallow-water environments; and (2) do not depend on sulfide. A similar symbiosis, regarding habitat and ecology, is the association between *Z. ignavum* and its ectosymbiont *Candidatus* Navis piranensis. Besides their presence on little degraded sunken wood [32], we also observed *Z. ignavum* and *Ca*. Navis piranensis inhabiting inorganic substrates such as plastics as well as various organisms. Additionally, we found the *Z. ignavum* symbiosis in the northern Adriatic Sea both in summer and winter. In contrast, the symbiosis between *Z. niveum* and its ectosymbiont *Ca*. Thiobius zoothamnicola, only thrives on substrates composed of or located near decaying organic matter [21–23, 31, 90–92]. *Z. niveum*’s ectosymbiont depends on sulfide, released during degradation of organic matter [73], as an electron donor as well as oxygen as electron acceptor [23, 24] to energize inorganic carbon fixation [25]. Additionally, *Z. niveum* and its ectosymbiont have so far been detected in temperate waters only during summer [23–25, 92], as the availability of sulfide in the ocean is strongly temperature dependent [93, 94] In the tropics and subtropics, the symbiosis can be found throughout the year [22, 31, 77, 90, 91].

The cultivation experiment, in which countless symbiotic *Z. mariella* sp. nov. colonies grew on a glass surface in untreated seawater, ultimately demonstrated that this symbiosis prospers under oxic conditions without a source of sulfide. A further *Zoothamnium*-bacteria symbiosis cultivable in steady flow–through systems is the thiotrophic ectosymbioses between *Z. niveum* and *Ca*. Thiobius zoothamnicola. In their 1996 redescription of *Z. niveum*, Bauer-Nebelsick et al. have already mentioned that the ectosymbiotic bacteria are probably sulfide oxidizers. They based their conclusion on the ectosymbionts’ conspicuous white colour, indicative of inclusions of elemental sulfur, and the presence of ribulose-l,5-bisphosphate carboxylase oxydase, a key enzyme in the Calvin-Benson cycle [31]. This led Rinke et al. to add sulfide to the seawater, when first cultivating the symbiosis in order to explore its growth requirements in 2007 [23]. Similar cultivation experiments combined with NanoSIMS and tissue autoradiography ultimately revealed that *Z. niveum*’s ectosymbionts indeed oxidize sulfide to fix inorganic carbon and subsequently transfer organic carbon to their host [25]. However, in the *Z. mariella* sp. nov. symbiosis a different approach is required, as there are no indications of potential sources of nutrition. To elucidate the symbiont’s and host’s nourishment as well as potential metabolic interactions, future studies can now be directed towards investigating the gene expressions of the two partners applying ‘omics’ techniques. Additionally, their interdependence could be explored by investigating the effects of antibiotics on their fitness.

## Supporting information

S1 Fig Epifluorescent micrographs showing unspecific bacterial epigrowth on the basal part of the stalk of large *Zoothamnium mariella* sp. nov. colonies. a, d) DAPI (blue), b, e) EUB mix in Cy5 (green), c, f) probe ZMS152 and ZMS1239 in Cy3 (red) did not hybridize.
(TIF)

S2 Fig Differential interference contrast (DIC) micrographs of living *Zoothamnium mariella* sp. nov. colonies with bacterial coat. a) branch with microzooids and a terminal zooid, b) microzooid, c) branch. mi: microzooid, t: terminal zooid, br: branch.
(TIF)

S1 Table Measurements taken from micrographs of living *Zoothamnium mariella* sp. nov. colonies. (XLSX)

S2 Table Morphometric characterization of *Zoothamnium mariella* sp. nov. zooids based on micrographs of living specimens. (XLSX)

S3 Table Morphometric characterization of *Ca*. Fusimicrobium zoothamnicola based on SEM-micrographs. (XLSX)
