# Supplementary figures and images for: Zoothamnium mariella sp. nov., a marine, colonial ciliate with an atypcial growth pattern, and its ectosymbiont Candidatus Fusimicrobium zoothamnicola gen. nov., sp. nov
Source: PLoS One. 2024 Apr 1;19(4):e0300758. doi: 10.1371/journal.pone.0300758 (PMC10984469; doi:10.1371/journal.pone.0300758)

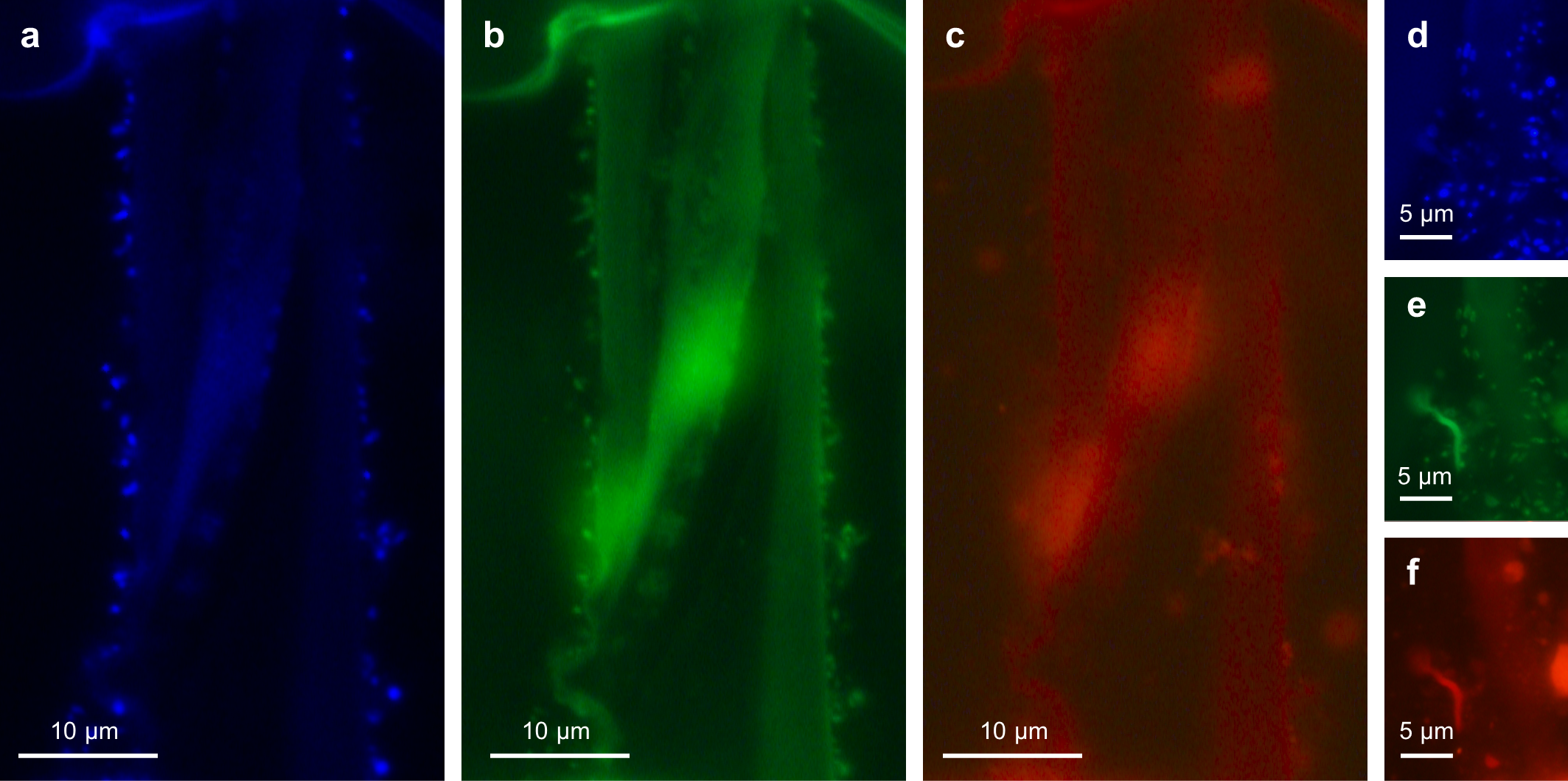

Supplement: S1 Fig — a, d) DAPI (blue), b, e) EUB mix in Cy5 (green), c, f) probe ZMS152 and ZMS1239 in Cy3 (red) did not hybridize. (TIF) [file pone.0300758.s001.tif]

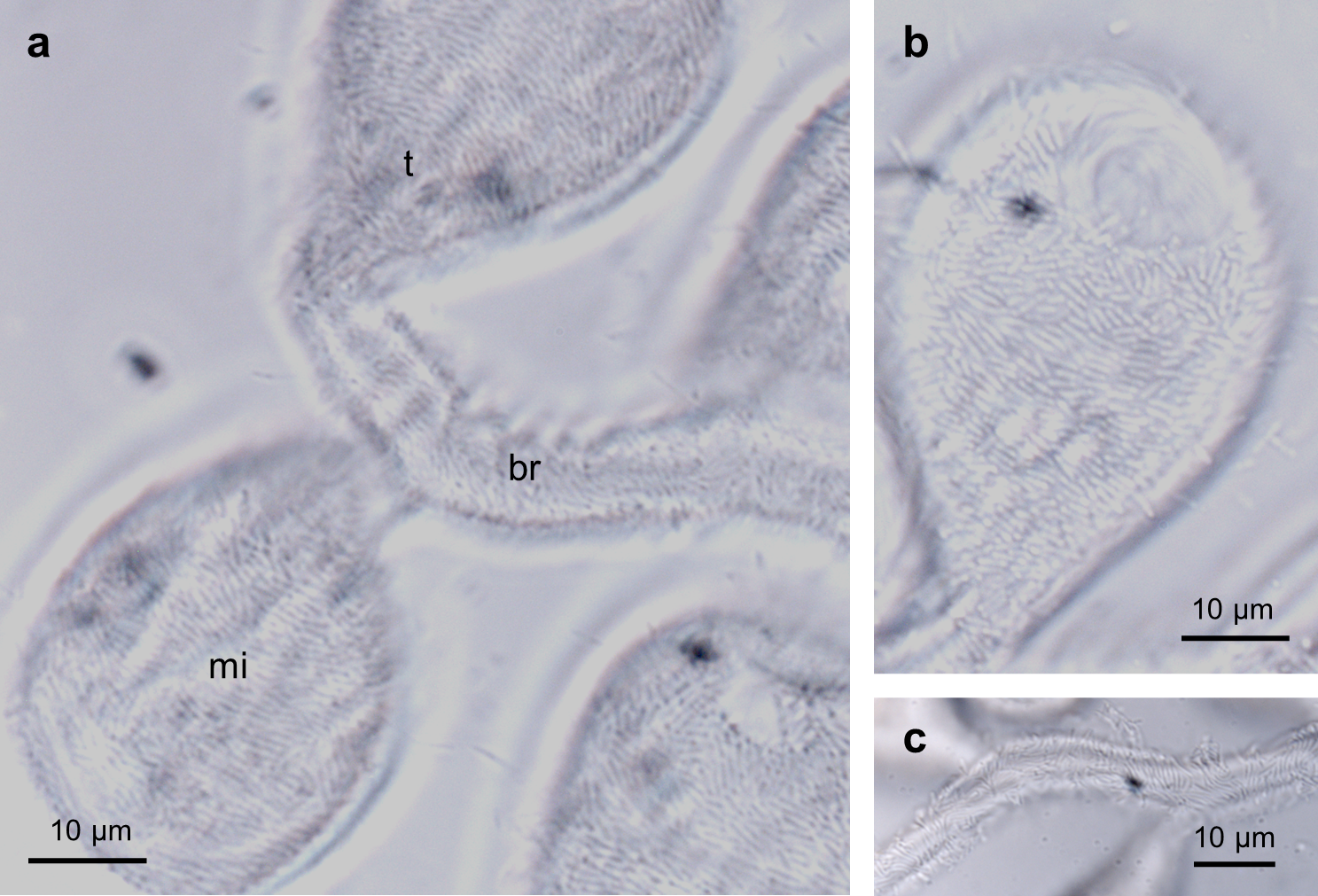

Supplement: S2 Fig — a) branch with microzooids and a terminal zooid, b) microzooid, c) branch. mi: microzooid, t: terminal zooid, br: branch. (TIF) [file pone.0300758.s002.tif]
